# Descriptive Epidemiology and Underlying Psychiatric Disorders among Hospitalizations with Self-Directed Violence

**DOI:** 10.1371/journal.pone.0059818

**Published:** 2013-03-26

**Authors:** Natalya S. Weber, Jared A. Fisher, David N. Cowan, Teodor T. Postolache, Rakel A. Larsen, David W. Niebuhr

**Affiliations:** 1 Preventive Medicine Program, Walter Reed Army Institute of Research, Silver Spring, Maryland, United States of America; 2 Allied Technology Group, Inc., Rockville, Maryland, United States of America; 3 Mood and Anxiety Program, Department of Psychiatry, University of Maryland School of Medicine, Baltimore, Maryland, United States of America; 4 Department of Preventive Medicine and Biometrics, Uniformed Services University of Health Sciences, Bethesda, Maryland, United States of America; University of Medicine & Dentistry of NJ - New Jersey Medical School, United States of America

## Abstract

**Background:**

Suicide claims over one million lives worldwide each year. In the United States, 1 per 10,000 persons dies from suicide every year, and these rates have remained relatively constant over the last 20 years. There are nearly 25 suicide attempts for each suicide, and previous self-directed violence is a strong predictor of death from suicide. While many studies have focused on suicides, the epidemiology of non-fatal self-directed violence is not well-defined.

**Objective:**

We used a nationally representative survey to examine demographics and underlying psychiatric disorders in United States (US) hospitalizations with non-fatal self-directed violence (SDV).

**Method:**

International Classification of Disease, 9^th^ Revision (ICD-9) discharge diagnosis data from the National Hospital Discharge Survey (NHDS) were examined from 1997 to 2006 using frequency measures and adjusted logistic regression.

**Results:**

The rate of discharges with SDV remained relatively stable over the study time period with 4.5 to 5.7 hospitalizations per 10,000 persons per year. Excess SDV was documented for females, adolescents, whites, and those from the Midwest or West. While females had a higher likelihood of self-poisoning, both genders had comparable proportions of hospitalizations with SDV resulting in injury. Over 86% of the records listing SDV also included psychiatric disorders, with the most frequent being affective (57.8%) and substance abuse (37.1%) disorders. The association between psychiatric disorders and self-injury was strongest for personality disorders for both males (OR = 2.1; 95% CI = 1.3–3.4) and females (OR = 3.8; 95% CI = 2.7–5.3).

**Conclusion:**

The NHDS provides new insights into the demographics and psychiatric morbidity of those hospitalized with SDV. Classification of SDV as self-injury or self-poisoning provides an additional parameter useful to epidemiologic studies.

## Introduction

Suicide claims over one million lives worldwide each year [1]. In the United States, suicide rates have remained relatively constant over the last 20 years (1/10,000 yearly), and risk factors, such as old age, male gender, gun access, stressful life events, and substance abuse have been identified [2]–[6]. More than 90% of those who die by suicide have at least one diagnosed psychiatric disorder, with depression and substance abuse disorders being the most common [7]. Among those with certain psychiatric disorders, the lifetime risk of suicide can be as high as four to seven percent, with nearly 30% attempting suicide [8], [9].

There are nearly 25 suicide attempts for each suicide, and previous suicidal and non-suicidal self-directed violence is a strong predictor of death from suicide [3], [10]–[13]. While many studies have focused on suicides, the epidemiology of non-fatal self-directed violence is not well defined. Because hospitalizations with self-directed violence are an important target population for preventive measures, it is crucial that we understand the population and risk factors that may have contributed to their hospitalization.

### Aims of the Study

In this cross-sectional study, we examined psychiatric disorders and demographic characteristics of hospitalizations with diagnoses of non-fatal self-directed violence. An emphasis was placed on identifying risk factors for self-directed violence resulting in injury, which may reflect a higher degree of intent to die, compared to poisoning. To our knowledge, this is the first study examining psychiatric disorders and methods of non-fatal self-directed violence in a sample representative of all US hospitalizations.

## Methods

### Data Source

The National Hospital Discharge Survey (NHDS) is conducted annually by the National Center for Health Statistics [14]. It uses a stratified three-stage probability design which covers discharges from short-stay, non-institutional hospitals located in all states and the District of Columbia, USA. Primary sampling units (PSUs) consisting of counties, groups of counties, or county equivalents comprise the first stage. In stage 2, hospitals within the PSU’s are selected with probabilities proportional to their yearly number of discharges. Stage 3 selects discharges by a systematic random sampling technique [15]. This enables national estimates of patients’ demographic characteristics, length of stay, diagnoses, and procedural codes in hospitals located in various regions of the country [16]. The 1979–2006 multi-year public use data and documentation files have standardized coding which provide comparable data across all data years. We have limited this dataset to the last 10 years available (1997–2006) in order to minimize differences in the utilization of International Classification of Diseases, 9th Edition/Revision (ICD-9) codes over time. The Walter Reed Army Institute of Research Institutional Review Board approved this study.

### Population

Demographic characteristics and analyses are presented for the total estimated number of discharges (285 million) based on the sample of 2,516,113 weighted records. Because our study examines and compares a disease burden in the hospitalized population, the discharge records associated with normal childbirth (ICD-9 code V27) were excluded. Only those aged 6 years or older and discharged alive were included.

### Data Coding

We use the term ‘self-directed violence’ or ‘SDV’ to replace the analogous ICD-9 E95 code definition of ‘suicide and self-inflicted injury.’ This terminology has recently been developed by the US National Center for Injury Prevention and Control in order to promote and improve the consistency of suicidal behavior surveillance and research [17]. Because there were few (N = 105) with SDV that were dead on admission or discharge, these hospitalizations were excluded from the study. Results are therefore presented only on those hospitalized with non-fatal SDV.

Hospitalizations with SDV were ascertained based on at least one supplementary ICD-9 code E950–E959 (suicide and self-inflicted injury) in the NHDS records, which lists between one and seven diagnoses. This section of the ICD-9 covers ‘injuries in suicide and attempted suicide’ and ‘self-inflicted injuries specified as intentional.’ SDV by poisoning was indicated by codes E950 (‘solid or liquid substances’), E951 (‘gases in domestic use,’ e.g., gas distributed by pipeline), or E952 (‘other gases and vapors,’ e.g., motor vehicle exhaust gas), while SDV resulting in injury was specified by codes E953 (‘hanging, strangulation, and suffocation’), E954 (‘submersion’), E955 (‘firearms, air guns, and explosives’), E956 (‘cutting and piercing instrument’), E957 (‘jumping from high places’), or E958 (‘other and unspecified means’). If a discharge record had E95 codes in both categories, the record was classified as SDV resulting in injury. Records with an ICD-9 code 290–319 in any diagnostic position were classified as having an underlying psychiatric disorder. Further classifications into general psychiatric disorders were also considered for analysis. These categories included: psychotic (ICD-9 290–295, 297–299), affective (ICD-9 296, 311), adjustment (ICD-9 309), anxiety (ICD-9 300), personality (ICD-9 301), and substance abuse disorders (ICD-9 303–305) [18].

### Statistical Analysis

The statistical analyses were performed using SAS software (version 9.2, SAS Institute Inc, Cary, NC, USA). The NHDS variable “weight” was used to provide national estimates (weighted frequencies) for demographic characteristics, region, source of payment, and length of stay for hospital discharges. Because some values for race were not recorded until beginning in 2000 and others too small to weight, we limited the reported values to ‘Black,’ ‘White,’ ‘Other,’ and ‘Not Stated.’ Ethnicity was not available. We calculated proportions (%) of discharge records with SDV among all discharges and compared them by demographic groups. Odds ratios from logistic regression with 95% confidence intervals (CI), adjusted for sex, age, race, region, and year, were used as measures of comparison and significance.

Time trends examining the counts and rates of SDV resulting in poisoning (E95.0–E95.2) or injury (E95.3–E95.8) were examined over the time period. Rates were estimated using US census data. Mean days of hospital care were also compared between discharges with indications of either injury or poisoning.

Proportions of psychiatric disorders among the hospitalized with and without SDV were calculated and compared stratified by gender. The proportion of discharge records that lists SDV resulting in injury was also presented for each grouping of psychiatric disorders. Discharges with SDV were further examined on the four and five digit ICD-9 levels to explore the most common methods of self-harm and their distribution between genders.

## Results

**Figure 1:**
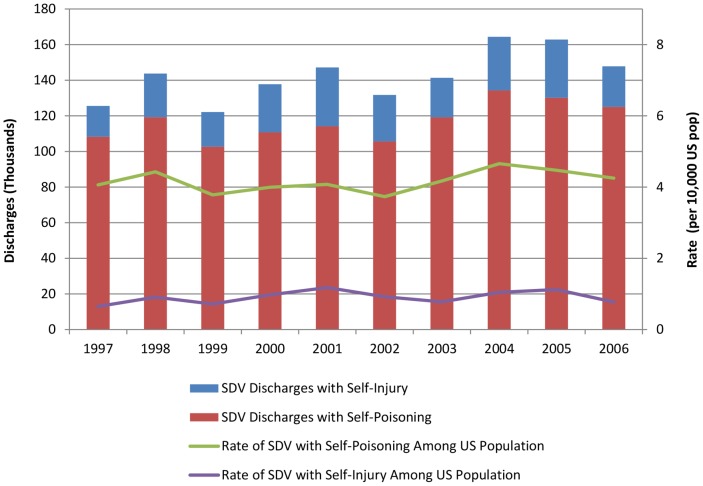
Counts and rates of self-directed injury and self-directed poisoning 1997–2006. SDV; self-directed violence.

The NHDS sample included 10,908 records with SDV, 0.5% of all records in the sample, representative of 1.4 million total hospital SDV discharges during the study period. The proportion of discharges with SDV remained relatively stable, with 4.5 to 5.7 hospitalizations per 10,000 persons per year (Figure 1). The rate of discharges with self-poisoning was roughly four times that of SDV resulting in injury, but neither rate meaningfully changed over the time period. Mean length of stay of inpatient care among discharges with SDV also did not change substantially over the time period but did differ significantly between injury and poisoning (5.6 days for self-injuries; 2.9 days for self-poisonings, *p*<0.001).

Important differences in the risks of SDV in general and among the subset of SDV resulting in injury are presented in Tables 1 and 2. Overall, there were nearly 300,000 more discharges with SDV among females than among males, due to the higher proportion of female discharges with poisoning codes (63%) (Table 1). While males were almost twice as likely to have SDV resulting in injury than in poisoning (OR = 1.96) (Table 2), there were roughly equal numbers of male and female discharges with self-directed injury. Hospitalizations with SDV were more likely to be younger individuals, especially adolescents, compared to those 65 and older. Whites were almost twice as likely as blacks to be hospitalized with SDV. The Midwest and West had higher proportions of hospitalizations with SDV.

**Table 1:** Demographic characteristics of hospital discharges with non-fatal self-directed violence (SDV) resulting in injury or poisoning.

		Proportion of Hospital Discharges with SDV	SDV Discharges with Self-Injury		SDV Discharges with Self-Poisoning	
		%	N	%	N	%
**Sex**	Female	0.54	122,330	47.9	727,726	63.0
	Male	0.44	132,852	52.1	427,397	37.0
**Age**	6–12	0.07	2,270	0.9	6,691	0.6
	13–19	2.78	57,229	22.4	212,572	18.4
	20–40	1.59	125,859	49.3	576,249	49.9
	41–65	0.43	58,772	23.0	319,759	27.7
	65–99	0.04	11,052	4.3	39,852	3.4
**Race**	Black	0.38	17,954	7.0	106,861	9.2
	White	0.50	152,388	59.7	745,450	64.6
	Other	0.50	10,408	4.1	42,702	3.7
	Not Stated	0.55	74,432	29.2	260,110	22.5
**Region**	Northeast	0.39	44,559	17.5	199,488	17.3
	Midwest	0.56	76,635	30.0	290,608	25.1
	South	0.44	72,274	28.3	394,951	34.2
	West	0.69	61,714	24.2	270,076	23.4

**Table 2:** Associations of demographic characteristics in hospital discharges with non-fatal self-directed violence (SDV).

		SDV vs. All Hospitalized		Self-Injury vs. Self-Poisoning		
		OR*	95% CI	OR*	95% CI	
**Sex**	Female	REF		REF		
	Male	0.82	0.77–0.88	1.96	1.65–2.32	
**Age**	6–12	4.00	2.66–6.02	1.34	0.54–3.29	
	13–19	76.43	63.06–92.64	1.06	0.67–1.70	
	20–40	43.38	36.05–52.19	0.80	0.51–1.25	
	41–65	11.39	9.42–13.77	0.68	0.42–1.09	
	> = 65	REF		REF		
**Race**	Black	REF		REF		
	White	1.82	1.63–2.03	1.23	0.92–1.66	
	Other	1.27	1.06–1.51	1.35	0.86–2.13	
	Not Stated	1.56	1.39–1.76	1.65	1.21–2.25	
**Region**	South	REF		REF		
	Northeast	0.94	0.86–1.03	1.15	0.90–1.47	
	Midwest	1.46	1.34–1.59	1.25	0.97–1.61	
	West	1.68	1.53–1.84	1.15	0.89–1.49	

* Adjusted for sex, age, race, and region.

**Figure 2:**
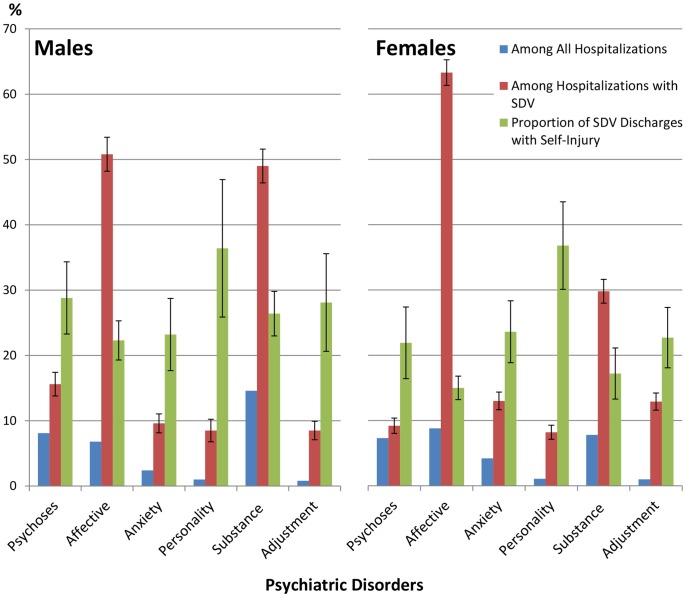
Relationship between psychiatric disorders and non-fatal self-directed violence in hospital discharges. The first two columns of each set represent the proportion of discharges with psychiatric disorders that occur among all hospitalizations or among all SDV discharges, respectively. The third column relates to the method of SDV if a discharge record has the given comorbid psychiatric disorder. The column presents the percentage of SDV discharges with self-injury as opposed to self-poisoning. Error bars are shown if the 95% CI for the weighted sample exceeds 2%.

Over 86% of the records that listed SDV also included ICD-9 codes for psychiatric disorders. The most common psychiatric disorders associated with SDV were affective disorders (57.8%) and substance abuse disorders (37.1%). Overall, there was a strong association between the co-occurrence of psychiatric disorders and suicide attempts (OR = 14.5; 95% CI 13.2–15.9). The association was strongest for those with affective (OR = 10.4; 95% CI 9.7–11.1) or adjustment (OR = 6.2; 95% CI 5.6–7.0) disorders. Comorbidity differed by sex (Figure 2). The co-occurrence of substance abuse disorders (OR = 2.2; 95% CI 1.9–2.5) or psychoses (OR = 1.7; 95% CI 1.4–2.1) was more common in males, while females were significantly more likely to have affective (OR = 1.7; 95% CI 1.5–1.9), adjustment (OR = 1.5; 95% CI 1.2–1.8), or anxiety (OR = 1.4; 95% CI 1.1–1.7) disorders. Discharge records with any psychiatric disorder category, except affective disorders, were more likely to also list SDV resulting in injury compared to self-poisoning. The association with self-injury was strongest for personality disorders compared to all other psychiatric disorders in both males (OR = 2.1; 95%CI 1.3–3.4) and females (OR = 3.8; 95% CI 2.7–5.3).

In all cases, ICD-9 E95 codes were detailed to the fourth or fifth digit, allowing an examination of the methods used for SDV. The majority of E95 codes indicated poisoning, most commonly by solid or liquid substances (81.5%). Among poisonings, the most frequently used substances were ‘tranquilizers and other psychotropic medications’ (37.1%) followed by ‘analgesics, antipyretics, and antirheumatics’ (26.1%). Cutting and piercing (E956) was the most common E95 code classified as a self-injury (11.3%). The distribution of percentages corresponding to method of self-harm differed between males and females. The male to female ratio was largest for discharges with SDV specific to firearms or explosives (OR = 17.9; 95% CI 7.7–41.3).

## Discussion

### Contribution to the Literature

Although data on suicides in the United States is well maintained by the National Center for Health Statistics, definitive data on non-fatal self-directed violence in general, or suicide attempts in particular, do not exist. Existing studies have primarily relied on self-reports on both national and smaller geographic scales [13], [19] to capture persons with a history of suicide attempt. To our knowledge, no study focusing on the hospitalized population with SDV has been conducted. Our study examines demographic characteristics and underlying psychiatric disorders in hospitalizations with SDV requiring hospital care in a large nationally representative sample free of self-reporting bias.

### Rate and Number of SDV Discharges

According to the CDC, the twelve-month incidence of suicide attempts is about 50 per 10,000 individuals [13]. Though not including non-suicidal SDV, this estimated rate is roughly 10 times greater than our finding. This, nevertheless, seems reasonable as our population does not include those attempts where medical care was not sought or did not lead to hospitalization. Doshi et al., approximated that, of 400,000 US yearly emergency department visits with attempted suicide or self-inflicted injury, roughly one-third result in hospital admission [20]. Our findings (of 140,000 hospitalizations per year) support this estimate and provide further insight into a population which includes those with SDV requiring hospitalization. The reasons for hospitalization include, but are not limited to, serious general medical consequences, continuous suicidal ideation, underlying psychiatric disorders requiring inpatient care, and/or the lack of adequate support and intervention efforts outside of the hospital.

### Demographics

It is important to note that the demographics of individuals who die by suicide differ dramatically from those with suicide attempts. For example, while males have a much higher suicide rate, females are at increased risk for suicide attempts. This gender difference in attempts versus suicides is one of the most widely supported findings in the epidemiology of suicidal behavior [21], [22]. Though the present study concerns only suicidal and non-suicidal SDV, we found that males were almost twice as likely as females to use means causing injury rather than poisoning. Though the proportions differed, there were roughly equal numbers of hospitalized males and females with self-directed injuries. Thus, it is important to keep both genders in mind when considering outreach, as individuals with self-directed injuries have a higher intent to die and a 2 to 5 fold increase in suicide risk compared to those with self-poisoning [10], [23]–[25].

In our population the number and proportion of discharges with SDV were higher among young compared with older individuals. Adolescents are especially vulnerable to stress and depression which may lead to higher rates of suicide attempts [26] and consequent hospitalization. Non-suicidal SDV is also a relatively common phenomenon in adolescence, mainly in those with various psychopathologies, and may require hospitalization. Although non-suicidal SDV usually contains no intent to die, it is associated with subsequent suicide attempts and may suggest that these behaviors and their related psychology may be a part of the same risk trajectory [11]. Unlike among adolescents, a higher proportion of older individuals die from suicide [27], [28] and therefore are not captured in our data. The elderly have a higher lethality from suicide because they use more lethal means [29], [30], more often live alone and therefore are less likely to receive prompt treatment after a suicide attempt, and their preexisting medical conditions also increase the likelihood of death [28].

### Psychiatric Comorbidity

The great majority of hospitalizations with SDV also had psychiatric disorders [2], [31]–[34]. Consistent with the literature, the major contributor to the SDV was a mood disorder with a more pronounced effect among women.

While the presence of an affective disorder was the strongest contributor to self-poisoning, a personality disorder was the highest risk factor for self-injury among both males and females. Using means resulting in injury could be attributed to a higher level of aggressiveness and impulsivity among those with personality disorders as well as more pervasive and more resilient psychopathology compared to persons with mood disorders [35], [36].

Among psychiatric hospitalizations the proportion of those with psychoses was substantial and similar for both genders. The proportion of underlying psychosis among discharges with SDV, however, was considerably higher in men compared to women. This gender difference could be attributed to a more severe course of schizophrenia and lower level of social functioning in men [37].

### Limitations

This study was limited by the inability to differentiate between suicide attempts and non-suicidal SDV based on the ICD-9 codes due to the lack of information on suicidal intent. However, we consider it important to study and appropriate to extrapolate our findings to and compare them with the existing literature on suicide attempts because many risk factors are shared by those involved in non-fatal SDV regardless of suicidal intent [38], [39]. Additionally, it is likely that many with SDV, even those that require hospital care, are missed or not coded properly by healthcare personnel [40], [41] thus underrepresenting the true number of those hospitalized with SDV. In our dataset, individuals could be included more than once if they had more than one hospitalization during the sampling period, therefore all findings are presented for hospital discharges and not individual patients. Although repeated SDV is common, multiple discharge records for one individual is unlikely given that only 1% of all hospital records have been sampled by NHDS. More up-to-date data would be preferable; however, the NHDS was discontinued after 2010 and the only multiyear datasets available to us did not extend beyond 2006. Breaking down racial groups more specifically and including ethnicity was also not possible due to lack of consistency and incidence within the dataset. Our study is observational and uses cross-sectional data, so no longitudinal information is captured on repeated SDV.

Some studies have broader criteria for identifying SDV to include ICD-9 codes E980–E989 (Injuries undetermined whether accidentally or purposefully inflicted) [34]. However we chose a more conservative approach and thus are more confident that all our cases presented with SDV, although, in return, we may have underestimated the frequency of SDV.

Our exploration of SDV hospitalizations resulting in either injury or poisoning is useful in understanding how SDV varies among demographic characteristics and among those with certain psychiatric disorders. This may better enable healthcare personnel to be cognizant of this high-risk population. Though demographics and characteristics of the population engaged in SDV differ in many ways, sometimes dramatically, from those that die by suicide, these persons are important as targets of intervention as they are at an increased risk for subsequent suicide.
